# Hand over Heart Primes Moral Judgments and Behavior

**DOI:** 10.1007/s10919-013-0170-0

**Published:** 2013-10-26

**Authors:** Michal Parzuchowski, Bogdan Wojciszke

**Affiliations:** 1 University of Social Sciences and Humanities, Sopot, Poland; 2 SWPS, ul. Polna 16/20, 81-745 Sopot, Poland

**Keywords:** Morality, Embodiment, Honesty, Cheating, Person perception

## Abstract

Morality is a prominent guide of both action and perception. We argue that non-emotional gestures can prime the abstract concept of honesty. Four studies demonstrated that the emblematic gesture associated with honesty (putting a hand on one’s heart) increased the level of honesty perceived by others, and increased the honesty shown in one’s own behavior. Target persons performing this gesture were described in terms associated with honesty, and appeared more trustworthy to others than when the same targets were photographed with a control gesture. Persons performing the hand-over-heart gesture provided more honest assessments of others’ attractiveness, and refrained from cheating, as compared to persons performing neutral gestures. These findings suggest that bodily experience associated with abstract concepts can influence both one’s perceptions of others, and one’s own complex actions. Further, our findings suggest that this influence is not mediated by changes in affective states.

## Introduction

Hand on heart, I did what I thought was right.

Tony Blair’s resignation speech

Most people think of themselves as highly moral (Wojciszke et al. 2011) though they commonly behave in a dishonest manner (Mazar et al. 2008). Yet, in many, if not most situations, people behave morally because they are motivated to remain true to their own norms and identities (Aquino and Reed 2002), or are reminded of their norms (Aquino et al. 2009), or they want to avoid aversive states such as dissonance resulting from the value-behavior discrepancy, social rejection and other punishment, or the mere self-categorization of oneself as a dishonest person (Mazar et al. 2008).

As discussed later, moral behavior is typically theorized as resulting from conscious deliberation, and when more automatic antecedents of morality are taken into account, they usually involve highly affective impulses. In the present line of studies, we theorize that both moral judgments and behavior may be driven by nonverbal embodied cues. These cues are non-affective in nature, yet increase morality, both in perception and behavior. Specifically, we identify putting the hand over the heart as a gesture emblematic of honesty, and we show in a line of studies that individuals performing this act are perceived by others to be more honest, and that persons unobtrusively performing this act themselves behave in a more honest way. We also attempt to show that these influences are not driven by changes in affective states which could possibly result from the hand-over-heart gesture. In effect, the studies reported here are the first demonstration that non-affective embodied cues can influence moral perceptions and behavior, without the mediation of affect.

## Morality

Morality, like language, is a human universal. Every known society possesses a moral code, though societies differ tremendously in the content and particular rules which determine the specifics of moral behavior. Moral systems are defined as “interlocking sets of values, virtues, norms, practices, identities, institutions, technologies, and evolved psychological mechanisms that work together to suppress or regulate selfishness and make cooperative social life possible” (Haidt and Kesebir 2010, p. 800). Because morality is a multidisciplinary subject of study, this definition surpasses the boundaries of psychology; however, from the point of view of social psychology, it implies that moral systems should be studied as regulators of both perception (and emotional experience), and behavior. Curiously, these two topics are rarely studied under the same theoretical auspices, or as a part of the same empirical program. Instead, studies of moral perception and moral behavior are studied as two distinct bodies of literature.

### Moral Judgment

The main debate on the nature of moral judgment has concerned the relative importance of reason versus emotion. According to the Kohlbergian (1984) rationalist tradition which dominated psychology for the second part of twetieth century (and which can be traced to Immanuel Kant), moral judgment relies on reasoning postulated to be context-independent and involves several steps in conscious, language-based thinking. In effect, forming a moral judgment is the process of uncovering a moral truth in a deliberate way. According to the emotionalist approach, moral judgments resemble instant perceptions rather than deliberate inferences, and the effect of these perceptions on judgment is mediated through emotional experience. Like other kinds of evaluations, moral judgments are frequently based on emotional intuitions (“gut feelings” of right or wrong), that emerge without intention or effort and they do so much more quickly than allowed by the assumption of a deliberate multistage processing. Support for the claim that at least some moral judgments are automatic can be found in experiments showing that judgments of morality emerge instantly, even when it is hard for the perceiver to supply them with any rule-related justification (Haidt 2001). Other studies have shown that procedural justice judgments are significantly based on the person’s own affective states in the absence of any rule-related information (Van den Bos 2003).

Although these two views of the nature of moral judgments are utterly discrepant, both enjoy substantial empirical support. There are at least two reasons for this paradox. First, rationalist and emotionalist approaches focus on situations involving moral judgments that differ in a number of important ways (Monin et al. 2007). Rationalist approaches ascertain the conclusion and resolution of complex moral dilemmas before any action is taken. An example of this is the Kohlbergian character Heinz, who is asked whether he should steal a drug to save his wife, or if he should refuse to break the law, and therefore let her die. By taking such hypothetical decisions as a prototypical case of moral situations, and then performing lengthy structured interviews with their participants, rationalistically-oriented researchers quite naturally find that participants engage in thoughtful deliberation when faced with moral situations, and logically conclude that moral judgment is based on complex reasoning. On the other hand, emotionalist approaches typically study responses to completed actions which violate moral norms, such as the case of consensual sex between adult siblings. Such blatant violations are bound to prompt quick emotional responses, leading researchers to the conclusion that moral judgment is typically affect-driven and rather lacking in rational thought.

The second reason why both approaches receive empirical support is that they tap into different psychological process (Haidt and Kesebir 2010; Greene 2007). Whereas moral reasoning draws on conscious, slow, and effortful information-processing, emotional responses tend to draw on processes which are automatic, fast, and effortless (Conway and Gawronski 2013; Gawronski and Bodenhausen 2007; Greene et al. 2008, 2004). There are many instantiations of dual-process models in social psychology, and most of them agree that automatic processes are always active, while deliberative processes are active only when the individual is both motivated to engage in and capable of conscious responding. Moreover, these two systems employ different relations between their elements when storing information and producing judgments. According to the reflective-impulsive model (Strack and Deutsch 2004) from which we generate our hypotheses, the conscious (reflective) system employs semantic relations represented in a propositional format (e.g., Jack is honest), and produces judgments via syllogistic inferences (Jack would not cheat). On the other hand, the automatic (impulsive) system is based on associations which result from contiguity and similarity, and generate affective or non-affective feelings based on spreading activation between associated elements. So, for example, if Jack is associated with honesty, then his mere appearance activates the honesty concept, and his dubious behavior will be not interpreted as cheating.

Everyday moral judgments frequently take yet another form—that of ascribing moral traits to the observed person (this person is unfair or honest, etc.). Although moral trait inferences are not typically classified as moral judgments, there is no doubt that such traits are frequently ascribed to others, and they profoundly influence both impressions and interpersonal behavior. In a classic study, Anderson (1968) measured the favorability of 555 traits; these traits covered all facets of personality, and the study found honesty and sincerity to be the most favorable of all traits (being ‘phony’ and ‘a liar’ were the most unfavorable). When the behavior of others is construable, both in moral and competence-related terms, people tend to construe the behavior predominantly in terms of morality. As such, moral traits are relatively more accessible, and global impressions of others are much more influenced by morally relevant information than competence-relevant information, even if the two are equal in evaluative extremity (Wojciszke 2005a). The morally relevant behavior of others leads to much stronger affective responses than do their competence-relevant acts (successes or failures), and moral relevance is the main predictor of the global evaluative meaning of the traits (Wojciszke 2005b).

Like other kinds of judgment, moral trait inferences may be products of either the reflective or impulsive system of information processing. On the one hand, numerous studies—inspired by classical attribution theory and other theoretical models (e.g., Srull and Wyer 1989)—showed that traits are inferred in conscious and purposeful ways from observed behavior, and circumstantial information. On the other hand, several paradigms show that traits may also be inferred unintentionally and without awareness. For example, in studies on spontaneous trait inferences, participants read sentences describing persons who performed actions that implied traits (e.g., “The child tells his mother that he ate the chocolates”). Later recall of the sentences was highest when cued by trait names (e.g., honesty), even when the traits in question were unmentioned in the original descriptions, and when the traits activated in this way primed subsequent perceptions (Uleman 1999). This suggests that traits are inferred very early on and quickly in person perception processes as a part of the comprehension of behavioral meaning. Similarly, traits are inferred from unknown faces in less than a 0.1 s (Todorov et al. 2009), a period of time that is certainly too short to allow for thoughtful deliberation.

### Moral Behavior

Large-scale studies have shown that behaviors pertaining to fairness and individual harm/care are morally relevant for most people, and for some (e.g., individuals belonging to collectivistic cultures or possessing right-wing political views), the in-group loyalty, deference to authority, and striving for purity are moral issues as well (Graham et al. 2009). This makes the category of moral behavior bewilderingly broad, as it includes norm maintenance and norm breaking, aggression and helping, cooperation and competition in social conflicts, as well as much in-group and inter-group behavior. It is hard to generalize across such a broad area of research, and no unitary theory of moral behavior exists. It is worth noting, however, that dual-process theories offer some prospects of a synthesis by suggesting that moral behavior—much like moral judgments—can result from either a reflective or impulsive system.

There is general agreement that human experience and behavior typically result from an interplay of two systems: an automatic, fast, and effortless one (the impulsive system), and a controlled, slow, and effortful one (the reflective system) (Chaiken and Trope 1999; Kahneman and Frederick 2002; Strack and Deutsch 2004). Dual process approaches have led to fruitful behavioral insights in virtually every field of social psychology, though there are some intriguing gaps. Specifically, our review of the literature on morality shows that the existing models and research tend to assume and find that immoral behavior may be both automatic and thoughtful, but moral behavior is typically considered to be thoughtfully reasoned.

Aggression is a good example of immoral behavior shown to be regulated both impulsively and reflectively (Berkowitz 2008). Research on reactive (provoked) aggression has shown that this impulsive behavior is essentially an unrestrained action, accompanied by rushed and insufficient reflection (traditionally called a lack of cognitive control). Both hostile perceptions and motives leading to such aggression may be primed even in a subliminal way, which results in hostile behavior; this behavior is exhibited without awareness of either the primes or their influence on the subject’s behavior (Todorov and Bargh 2002). The depletion of an individual’s ability to utilize thoughtful self-control leads to an increase in provoked aggression, but the restoration of the person’s self-control results in decreased aggression (Denson et al. 2011). Still, there are numerous cases of aggression being deliberative in nature: aggression can change based on the individual’s understanding of provocation, or on the predicted consequences of one’s own behavior, and the aggressive behavior may be planned well ahead of the aggressive act actually being carried out.

On the other hand, moral behavior is typically—though not always—portrayed as deliberative. For example, studies inspired by the social cognitive model of moral behavior (Aquino et al. 2009) found that people behave in moral ways (e.g., refrain from lying even when they would benefit from doing so) based on to which degree their working-self includes moral norms. This in turn depends on the chronic and situational differences in the accessibility of one’s moral identity. For some people, their moral identity is central and chronically accessible, which strengthens their motivation to act in line with their moral norms. For others, their moral identity is inaccessible unless it is activated by situational factors, and thereby made a part of the conscious working-self. This may be accomplished in such ways as recalling the Ten Commandments, or writing a story using morally-laden terms. Moral identity may also be deactivated by the presence of financial incentives which lead to a decrease in moral motivation and behavior. Generally, people consider themselves highly moral (Wojciszke et al. 2011), yet they strive to maximize financial benefits which result from dishonest behavior. When tempted to acquire benefits through cheating, people only cheat enough to receive about 20 % of the possible benefits maximum. This is behaving “dishonestly enough to profit, but honestly enough to delude themselves of their own integrity” (p. 633), as Mazar et al. (2008) put it. These authors have shown that such maneuvers disappear when moral norms are brought to an individual’s conscious attention, thereby encouraging people to behave in honest ways. Moreover, depleting self-control resources increases a person’s yielding to temptation, which leads to cheating; this link is mediated by an impaired ability to recognize the immorality of the act of cheating. Resisting unethical temptation both requires and depletes self-control resources (Gino et al. 2011).

To summarize, research on moral behavior typically conceptualizes immoral behavior as the product of both impulsive and deliberative acts, while moral behavior is thought to be driven mostly by deliberation. Even empathically driven helping—which comes to mind as an example of impulsive moral behavior—actually requires a substantial amount of conscious deliberation. Empathic helping requires perspective-taking; this is a threshold function of one’s ability to take another person’s perspective into account. In effect, perspective-taking is exhibited in cases “in which we try to imagine how the person in need is affected by his or her situation” (Batson and Shaw 1991, p. 112). Nevertheless, automatic reactions of empathy are observed for highly emotional stimuli like people experiencing physical pain (Decety and Lamm 2006; de Vignemont and Singer 2006; Singer and Leiberg 2009). Also, the experience of synchronizing one’s movements with those of another person leads to inferences of similarity and compassion resulting in altruistic behavior directed toward the person (Valdesolo and DeSteno 2011), and plausibly it is a result of automatic rather than deliberative processes. Given such data, and the fact that impulsive processes and representations are basically associational and not necessarily egoistical in nature (as discussed later), the relative dearth of studies on the impulsive antecedents of moral behavior may simply be a gap in the existing research, rather than a theoretical impossibility. In the present line of studies, we aim to fill this gap and to show that the gesture of placing a hand over the heart can serve as an impulsive antecedent of both moral judgment and behavior.

## Present Studies

When discussing the ‘reason versus emotion’ debate in moral judgment, Haidt (2001) points out that human emotions are usually so saturated with cognition that it is better to speak of ‘moral reasoning versus intuitions’, and to conceptualize both as cognitive responses. Whereas reasoning is both conscious and effortful, a moral intuition is “an evaluative feeling (like–dislike, good–bad) about the character or actions of a person without any conscious awareness of having gone through steps of search, weighing evidence, or inferring a conclusion” (Haidt and Bjorklund 2008, p. 188). Indeed, numerous studies find evidence of affective underpinnings of moral intuitions. Moral judgments are frequently influenced by the perceivers’ affective responses, which may have nothing to do with the moral meaning of the observed behavior. For example, the emotion of disgust induced by a filthy environment can be misattributed to unrelated context, and increase the severity of moral judgments people make afterwards (Schnall et al. 2008). As already mentioned, people are frequently able to form instant evaluations of moral transgressions, even if they are unable to provide rational reasons for such judgments afterwards (Wheatley and Haidt 2005). Finally, making moral personal judgments involves relatively greater activity in brain areas related to emotion (the ventro-medial prefrontal cortex), while impersonal moral judgments involve relatively greater activity in brain areas associated with cognitive processes (Greene et al. 2004). Damage of the VMPC deprives the individual of their intuitive feelings of right or wrong, as well as the downstream ability to behave accordingly (Greene 2007).

Does all this mean that each and every antecedent of moral intuition and action is affective in nature? Not necessarily, we think. Moral intuitions are products of the impulsive system which is based on associational architecture, and on spreading activation between the associated elements. These bidirectional associations can connect both associative and deliberative elements; these elements include concepts, feelings, motives, and behavioral schemata (Strack and Deutsch 2004). A classic example of an affective association is the link between vertical and horizontal head movements (nodding and shaking the head) and, respectively, feelings of agreement and disagreement. Participants unobtrusively induced to nod encoded positive information more so than negative information, while participants induced to shake their head encoded negative information more so than positive information (Förster and Strack 1996; Petty and Cacioppo 1983).

### Gesture of Honesty

Bodily sensations influence the way we think, feel, and act (Barsalou 2008; Niedenthal et al. 2005) through a variety of pathways, including the priming of related semantic concepts (Chandler and Schwarz 2009; Riskind 1983). Consequently, the mere experience of a bodily sensation may activate the associated concept; this in turn may shape information processing and behavior. Numerous studies have demonstrated that individuals’ thoughts, feelings, and behaviors are influenced by body movements (e.g., Mussweiler 2006; Strack et al. 1988), hand configurations (Schubert 2004), gestures (Chandler and Schwarz 2009), arm movements (Förster and Strack 1997), and head movements, as already mentioned.

Movements or gestures influence the activation of a concept, even when they are unobtrusively induced in such a way that their meaning is disguised from the participants’ awareness. For example, Chandler and Schwarz (2009) informed their participants that they were studying the influence of hand movement on text comprehension. Under this guise, they asked their participants to extend their middle finger (a gesture associated with hostility), or to extend their thumb upward (a gesture associated with approval), or to extend their index finger (a neutral, control gesture). While making the gesture, the participants read an ambiguous description of a person (Donald), and then indicated their impressions of the person. None of the participants were aware of the gestures’ purposes, nevertheless those making the hostility-associated gesture perceived the target person as more hostile than did the controls, and participants making the approval gesture perceived the target in a more positive way than did the controls.

The meaning of gestures used by Chandler and Schwarz is arbitrary and culture-specific. For contemporary Americans, “giving someone the finger” means hostility, but for Americans half-a-century ago, the gesture meant defiance (Chandler and Schwarz 2009). Is there a gesture culturally associated with honesty? The present investigation is based on the idea that a hand-over-heart gesture can prime honesty. Many cultures associate the gesture of placing a hand on one’s heart with honesty. The gesture indicates that one is not bearing arms, or that one appears to have genuine intentions, or is giving one’s word of honor, or is pledging allegiance (Eibl-Eibesfeldt 1996). Indeed, since the time of Aristotle (Bakalis 2005), people have believed that the heart is the seat of the human mind, and symbolically it is still used to refer to the emotional or moral core of human beings. Additionally, many languages (e.g., British-English, German, Polish, Russian, and Slovak) have idioms that express honesty through reference to the gesture of putting a hand on one’s heart. For example, people might say “from the heart” or “the heart of the matter” to suggest that their statements are honest. In Poland (where the present studies were conducted), “with hand over heart” (*z ręką na sercu*) is an idiomatic expression of honesty used at the end of any dubious statement, and the gesture is a common emphasis of sincere intentions. Thus, we expect this gesture to be associated with honesty.

### Hypotheses

On the operational level, we tested two simple hypotheses: (1) that persons making the hand-over-heart gesture are perceived to be more sincere and honest, and (2) when individuals are unobtrusively manipulated into making the hand-over-heart gesture, they behave more honestly. Hypothesis 1 may be seen as a mere extension of Chandler and Schwarz’s (2009) findings as related to another gesture, but we believe that it is actually a substantial extension of the existing studies on embodiment influences on perception. Whereas in all the studies discussed so far, the embodiment concerned the perceiver (the person who made the gestures, body movements, etc.), in our first two studies the embodiment concerns the perceived person. It is the perceived target, not the perceiver, who makes the hand-over-heart gesture. Confirming Hypothesis 1 would therefore suggest the crucial role of the mental representation of body states, not necessarily their experience. If the perceiver’s gestures influence his or her perceptions, we do not know whether it is the sensation of the gesture or its mental representation which counts. If, however, the gestures of the perceived person influence perception, it is much more likely that associations (accessible for the perceiver), rather than direct experiences (inaccessible for the perceiver), are responsible for this influence.

Hypothesis 2 concerns the influence of gestures on the behavior of the gesturing person. Confirming Hypothesis 2 would contribute to moral reasoning studies by identifying an additional associative source of influence. This would suggest that moral intuitions yield to non-affective influences, not only affective ones, as shown by existing studies (i.e., Zhong and Liljenquist 2006). This hypothesis also goes well beyond showing yet another culture-bound case of embodiment, as it demonstrates that gestures influence not only perceptions, but also related behaviors. Although concept activation is studied because behavior is assumed to follow from it, the link between stimuli and subsequent behavior is necessarily more tenuous than the direct effect of stimuli on perceptual experience and evaluations. This would contribute to the embodiment literature, because there are few examples of body movements influencing behavior, and those which do exist tend to focus on valenced evaluations of the self (Förster and Friedman 2008).

## Experiment 1

**Fig. 1:**
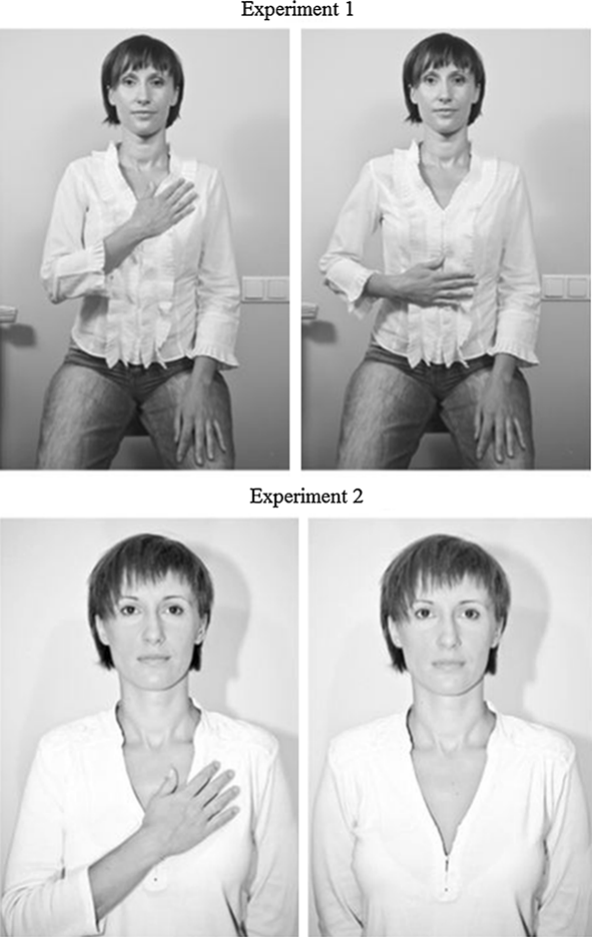
Photographs of targets used in Experiment 1 (*top panel*) and Experiment 2 (*bottom banel*)

First we wanted to test if hand-over-heart is an emblematic gesture among Polish speaking participants; essentially, we tested whether this particular hand movement can symbolically communicate honesty. If people associate the hand-over-heart gesture with honesty and telling the truth, they should infer traits associated with honesty when this gesture is seen, even if they do not explicitly think about the meaning of the gesture. To test this hypothesis, we asked participants for an open-ended description of a person performing the honesty gesture, and counted how many of traits used to describe the person pertained to honesty. Because traits related to honesty may be inferred from the facial features of target persons (not their gestures, cf. Todorov 2011), we also introduced a control condition with the target person placing her hand over her stomach. This gesture has an emblematic meaning (stomach ache) that is unrelated to honesty. The facial expression, posture and target’s lighting were identical across all the photographs (shown at the top panel in Fig. 1).

### Method

#### Participants and Design

One hundred and eighteen Polish participants (85 female; *M*_age_ = 26.2; *SD* = 6.76) completed this online study in response to an invitation published on a popular educational website. The study was presented as dealing with person perception, and the participants were asked to write a short description of a young woman showed in a photograph, either with her hand over her heart or with her hand over her stomach. Seventy-four out of the 107 people who visited the website completed the written description when the photograph depicted a hand-over-heart gesture (the completion rate was 69 %), while this was true for 44 out of 85 respondents when the control gesture was used (the completion rate was 52 %).

#### Procedure and Dependent Measure

The cover story presented the study as dealing with the communication skills of the photographed person. Participants were asked to answer a single question: “*What does the person in the photograph communicate to others? List 5 associations that come to mind when you see this person*”. Both conditions differed only in the randomized use of the gesture presented in the photograph. After listing traits associated with the target person, participants were debriefed and thanked for their participation. In accordance with the primacy-of-output method, a concept is considered accessible if it is mentioned as the first trait in the description (see Higgins et al. 1982; Narvaez et al. 2006). Therefore we analyzed the content of the 118 traits listed first by our participants (74 traits describing the person performing the hand-over-heart gesture, and 44 traits describing the person producing the hand-over-stomach gesture). Two independent judges (one female), blind to the hypothesis and condition, obtained a randomly-ordered list of all the traits and were asked to classify each dichotomously as pertaining to honesty or not (*this trait is related to honesty* vs. *this trait is not related to honesty*). Ratings from both judges were correlated (*r* = .68; *p* < .001), and we used an aggregate variable as an association to honesty index of each described trait.

### Results and Discussion

As predicted, a person performing the hand-over-heart gesture was described in terms pertaining to honesty by a greater number of participants than was the person in the control condition. Specifically, 49 % of participants (36 out of 74) used terms pertaining to honesty to describe the person performing the hand-over-heart gesture, while in the control condition, honesty was the first association for 18 % of participants (8 out of 44), χ^2^(1, 118) = 10.95; *p* = .001; *φ* = .30. This finding supports the hypothesis that merely seeing a person performing the hand-over-heart gesture activates the concept of honesty. This establishes a link between this gesture and perceived honesty. It clearly suggests that one can encode and efficiently communicate honesty without language, and by merely using an emblematic gesture.

## Experiment 2

Experiment 1 showed that the hand-over-heart gesture is seen as a signal communicating sincerity or honesty. However, is this signal believed and trusted, and does it give rise to inferences included in the spontaneous impressions of the signaler? Specifically, are the persons performing this gesture perceived as being more honest? To answer this question, we conducted a second study, one in which participants rated the truthfulness of a target person performing the hand-over-heart or a control gesture. To substantiate multiple queries about the target person’s credibility, we used the setting of a job interview, where an interviewee’s truthfulness is an issue that naturally arises. Our participants listened to an interviewee presenting herself orally while performing the hand-over-heart or a control gesture. Afterwards they rated the interviewee’s self-descriptions for credibility. We expected the interviewee performing the hand-over-heart gesture to be rated as more credible than the person performing a control gesture.

### Method

#### Participants and Design

Thirty-seven Polish university students (20 female; *M*_age_ = 20.08; SD = 2.08) listened to a fragment of a 4-min audio recording of a job interview. While listening, they looked at a photograph of a young female interviewee standing straight, with either both of her hands behind her back, or her right hand placed on her heart (bottom panel of Fig. 1).

The cover story presented the experiment as a study on the correlates of impression formation. Participants studied the photograph (with the target person performing either the honesty or control gesture), while listening to an audio recording that contained a short self-description. Among the claims made in the self-description were boastful statements taken from a Polish adaptation (Drwal and Wilczynska 1995) of the Social Desirability Scale (Crowne and Marlowe 1960) describing socially approved but highly improbable behaviors (“I have never been late for work”; “I never postpone anything to the future”, “I always keep my promises”, “I am kind to everyone”, “I always respond to letters”, “I have never cheated anyone”, “I have never called in sick” and “I have never argued with members of my family”*).* Each of these statements was rated on a 7-point scale (ranging from 1—*This* *is not credible at all,* to 7—*This* *is very credible*). The averaged answers to these questions resulted in a reliable index of the target person’s credibility (*α* = .74). Next, the participants were asked to describe the target’s physical appearance and then thanked for their participation. Importantly, in these post-experimental interviews, no participant mentioned the target’s gesture.

### Results

As predicted, the credibility of a female performing the hand-over-heart gesture was rated higher (*M* = 4.68; SD = .64) than the credibility of the same person with both hands behind her back (*M* = 4.17; SD = .79), *t* (36) = 2.17, *p* < .05, *d* = .70. This intertwines smoothly with the findings of Experiment 1, which showed that people perceive the hand-over-heart gesture as a signal of honesty. The present findings show that the signal is believed and spontaneously included in an impression of the signaler. A person producing this signal is perceived as more credible, even if her statements are not very credible (such as self-flattering statements made during a job interview). So, a performance of the hand-over-heart gesture increases the judgments of the honesty of the person who performs this gesture. In the next two studies, we turn to the hypothesis that an unobtrusive performance of this gesture also increases the moral behavior of persons who put their hands over their hearts.

## Experiment 3

Experiment 1 showed the prevalence and accuracy in reading the meaning of the hand-over-heart emblematic gesture, while Experiment 2 showed the actual incorporation of the gesture message into the judgment of its sender. To test whether the gesture can influence not only perception but also behavior, we turned to the question of whether performing the gesture increases the honesty in the performer’s own behavior. In this study, we focused on “white lies”, those small lies which smooth social interactions, and facilitate the maintenance of interpersonal relationships by downplaying negative attitudes (DePaulo 2004; DePaulo and Kashy 1998) and increasing prosocial behavior (DePaulo et al. 1996). We examined whether bodily primed honesty reduces the proclivity to tell white lies, thereby making people more honest (though less polite) in their assessment of others. Our participants were asked to rate the physical attractiveness of a set of young women; the women were introduced by the experimenter as his friends. The photos were preselected to include persons who were moderately attractive or extremely unattractive. We assumed that in such a situation, the participants would tend to downplay the extremity of their evaluations of the unattractive targets (“the experimenter’s friends”). We assumed this because, first, there is a general rule of politeness which forbids the negative evaluation of an interaction partner’s physical appearance. This rule may be observed from even very early in life—it has been observed, for example, as prevalent among preschoolers (Talwar and Lee 2002), and we assumed that this prohibition of negative evaluations spills over to other entities (like friends) associated with the partner. Second, we made this assumption because people tune their communicated opinions to the presumed attitudes of their audience. Higgins and Rholes (1978) showed the “saying is believing” effect. This effect suggests that after communicators learned whether their audience liked or disliked a person who was the target of communication, the communicators portrayed the target more positively to the audience who liked the target person, and more negatively to the audience who disliked them (cf. Echterhoff et al. 2008). Thus, it is a natural part of communication to be slightly insincere in conveying one’s own opinions as more congruent with the opinions of an interlocutor than they really are. If the hand-over-heart gesture promotes sincerity and honesty, the gesture should result in more sincere but less-flattering evaluations of the experimenter’s “friends” when compared to the control condition. This should be true for the evaluations of the experimenter’s extremely unattractive friends, but not for the ratings of the moderately attractive ones, because there is no motivation to lie about the latter’s appearance.

### Method

#### Participants and Design

Forty-eight right-handed Polish university students (40 females, *M*_age_ = 20.16; SD = 1.50) participated in the study in exchange for a course credit. Participants were randomly assigned to either make hand-over-hip (control group) or hand-over-heart (experimental group) gesture while judging the physical appearance of women who were presumably friends of the experimenter.

#### Stimulus Materials

Stimulus photographs were selected from a foreign (German) website where people submit their pictures to be judged by others (similar to www.hotornot.com). A preliminary group of 35 photographs was pretested by 16 judges (8 males) on a scale ranging from 1 (*definitely unattractive*) to 9 (*definitely attractive*). This yielded a set of 5 pictures of very unattractive women (*M* = 1.17, SD = .24) and 5 pictures of women of moderate attractiveness (*M* = 2.65, SD = .62).

#### Procedure and Dependent Measure

**Fig. 2:**
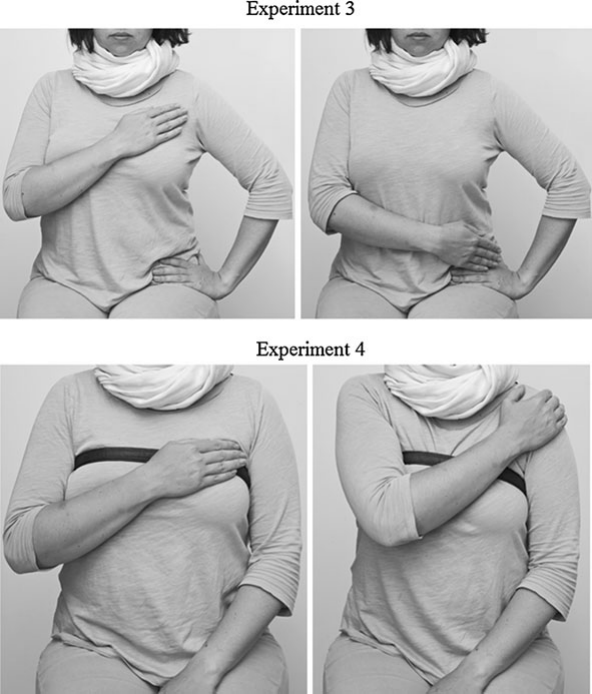
The gestures that participants were instructed to perform in Experiment 3 (*top panel*) and Experiment 4 (*bottom panel*)

As a part of the cover story, we informed our participants that the experiment dealt with the effects of cognitive load on judgments of appearance. The “cognitive load” instructions asked people to rate the pictures while performing the “parallel task” of making a specific physical gesture. Participants were asked to place their non-dominant hand on their left hip with their back straight. They were then asked to place and hold their dominant hand either 15 cm (6 inches) below the collar bone (resulting in the hand-over-heart gesture) or 15 cm below their last rib (resulting in them holding their hip, as shown at the top panel in Fig. 2). Participants held this posture while viewing the moderately attractive and extremely unattractive females in random order (for 10 s each). Participants were asked to form impressions of the faces, which were introduced as being taken from the social networking profiles of the experimenters’ long-time friends. This should have resulted in the activation of the politeness norm (being nice when talking about somebody’s friends). After the slideshow, participants (with their hands free) had a chance to briefly re-examine the faces and rate their attractiveness on a 9 point scale (ranging from 1—*This person is extremely unattractive*, to 9—*This person is extremely attractive*). After the procedure, participants were thanked and debriefed. Importantly, no participants guessed the correct hypothesis or mentioned anything about the idea of honesty, or the social meaning of the postures.

### Results and Discussion

**Fig. 3:**
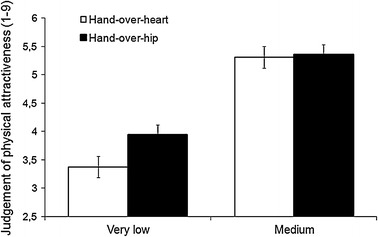
Experiment 3 Judgment of physical attractiveness provided by participants performing hand-over-heart and hand-over-hip gesture as a function of target’s appearance (faces of very low vs. medium attractiveness). *Error bars* represent 1 SEM

A 2 (low vs. moderate attractiveness; within subjects) × 2 (hand placement: heart vs. hip; between subjects) analysis of variance was performed on attractiveness ratings. This analysis revealed a main effect of attractiveness, with unattractive faces being rated much lower (*M* = 3.66; SD = .98) than those of moderate attractiveness (*M* = 5.34; *SD* = .84), *F* (1, 46) = 129.35, *p* < .001, *d* = 1.84. However, this effect was qualified by an interaction between physical attractiveness and hand placement, *F* (1, 46) = 5.00, *p* < .05, *η*_*p*_^2^ = .09 (see Fig. 3). A planned contrast revealed that unattractive faces were rated significantly lower by participants keeping their hand over their heart (*M* = 3.38; SD = 1.16) rather than on their hip (*M* = 3.95; SD = .66), *F* (1, 46) = 4.29, *p* < .05, *d* = .60. This difference was not observed for moderately attractive faces (*M*_*heart*_ = 5.31; *SD* = .67 vs. *M*_*hip*_ = 5.36; *SD* = 1.01), *F* < 1. Thus, when presented with an opportunity to lie about someone’s appearance, people who put their hands over their hearts remained more honest, even if it meant being impolite. Importantly, this interaction shows that the present results cannot be interpreted in terms of an increased positivity in evaluations resulting from the hand-over-hand gesture. Had this gesture operated via increases in affective positivity, ratings of both attractive and unattractive target persons would have been increased. In actuality, only the ratings of unattractive targets were influenced, and those ratings were decreased in the gesture condition, thereby ruling out the interpretation in terms of increased positive affectivity.

To conclude, the present study showed that people unobtrusively induced to make the hand-over-heart gesture behave in a more moral way. Because the predicted and obtained pattern of the results involves decreases in judgments of some (unattractive) target persons, it cannot be explained in terms of increased positivity of the affective state of persons performing the critical gesture (because this would require increases in judgments). In Experiment 4, we extend this result by examining whether people who make the hand-over-heart gesture also act in a more moral way by abstaining from cheating when given an opportunity to do so.

## Experiment 4

Because our idea that moral behavior can be automatically increased as an effect of non-emotional embodiment is a novel one, we decided to test it once more using a different kind of moral behavior as a dependent variable. In this study, we enabled our participants to cheat while performing the hand-over-heart gesture, and we expected them to cheat less than the participants who performed in the control condition, and who were given a test that was devised in a way which prevented cheating. We expected that people performing the hand-over-heart gesture would refrain from cheating.

Moreover, there is solid data showing that an inward (as opposed to outward) arm flexion results in a generally heightened affective state (Förster and Strack 1998; Priester et al. 1996), presumably due to its association with the approach system. Because our hand-over-heart gesture also involves an inward arm flexion, it could increase moral behavior via the intensification of positive affective states. To rule out this alternative interpretation of the expected results, we introduced an additional control condition with a gesture of hand-over-shoulder (the right hand resting on the clavicle bone of the left shoulder) that also involves an inward arm flexion, though this gesture is not associated with honesty. If the expected increases in honesty are due to an inward arm flexion, our participants should cheat less in both gesture conditions compared to a control, no-gesture control. If, however, the increases in honesty are due to the specific association of the hand-over-heart gesture with honesty (as shown in the previous studies), then our participants should cheat less in this condition compared to the other two conditions. Finally, we asked the participants in Experiment 4 to perform the gestures in a private setting; this is in contrast to the previous three studies, in which the participants performing the gestures were aware of being observed. In this way, we wanted to check whether the gesture-honesty link depends on the presence of observers (and possible self-presentational concerns), or not.

### Method

#### Participants and Design

Fifty-two Polish university students (34 female; age was not recorded) took part in the study which involved solving math problems. In the control group (*N* = 17), participants had to write down their solutions so there was no possibility of cheating. In the two experimental groups, the participants had their dominant hands occupied with an additional activity which prevented them from writing down the solutions. So, they were asked to just remember the number of solved problems and report them later, thereby allowing them to cheat by inflating the number of solutions reported. In the first experimental group, the additional activity involved the hand-over-heart gesture (*N* = 18), whereas in the second experimental group, the activity involved a neutral gesture of placing the hand on their shoulder (*N* = 18).

#### Procedure and Dependent Measure

The study was presented as a procedure to calibrate a new device designed to monitor breathing. Participants were asked to wear a “breathing monitor band” and hold it on their chest either with their right palm (which resulted in the hand-over-heart gesture), or with a forearm (which resulted in the neutral gesture of placing their right hand on their opposite shoulder, as shown at the bottom panel in Fig. 2). While the experimenter monitored the participants’ breath, the participants were asked to solve simple math problems. The problems consisted of 20 matrices, each containing 12 numbers aligned in 4 rows and 3 columns, and each requiring participants to find two numbers that would add up to 10 (adopted from Mazar et al. 2008). They were asked to solve as many matrices as they could in 5 min, while their breath was being measured. To enhance the motivation to cheat, we promised that after the experiment, one randomly selected participant would earn 5 Polish zlotys (~$2) for each matrix solved correctly. The participants were left alone to solve the task and as there was no evidence of their actual performance, they had ample opportunity to cheat. To compare the hand configuration manipulation conditions with a baseline level of performance on the search task, we also included a control group that had no respiration monitor installed, and had no opportunity to cheat because they were asked to write down their answers. After the procedure, participants were thanked and asked what they thought the study was about; none guessed the correct hypothesis, or mentioned the meaning of the gestures involved.

### Results and Discussion

A one-way ANOVA revealed a significant effect of the condition, *F* (2, 49) = 3.36, *p* < .05, *η*_*p*_^2^ = .12. When given the opportunity, participants in the hand-over-shoulder condition cheated more (*M* = 7.89; SD = 3.64) than those in the hand-over-heart condition (*M* = 5.50; SD = 3.36), planned contrast *t*(49) = 2.21, *p* < .05, *d* = .68. The former also reported more correct solutions than those in the baseline control condition (*M* = 5.37; SD = 2.52), *t* (34) = 2.25, *p* < .05, *d* = .80, with no significant difference between the hand-over-shoulder and the no-gesture condition (*t* < 1). While participants in the hand-over-heart condition behaved honestly (reporting the same amount of matrices solved as the control condition which did not allow cheating), participants in the shoulder condition cheated, claiming 45 % more solved matrices than the other two groups. Again, even the private and unobtrusive use of the hand-over-heart gesture serves as a cue for behaving in accordance with moral standards that are linked with the meaning of this hand placement. Moreover, this study ruled out the possibility that this effect is driven by increases in positive affective state which could result from an inward arm flexion involved in the hand-over-heart gesture.

## General Discussion

### Summary of the Findings

Our studies provide the first demonstration that honesty can be manipulated through non-affective associative cues. Taken together, these findings link embodied cues and honesty, demonstrating that both the perceived and realized levels of honesty can be manipulated through the unobtrusive performance of the hand-over-heart gesture. Persons photographed while making the hand-over-heart gesture appeared to signal honesty (Experiment 1), and were perceived as more credible, indeed, than the same persons performing a control gesture (Experiment 2), suggesting that this gesture is associated with honesty and is used as a cue to infer this trait. Furthermore, an unobtrusive performance of this gesture leads people to behave more honestly when evaluating the physical appearance of others (Experiment 3), and when tempted to cheat (Experiment 4). These effects cannot be explained in terms of the (presumably) more positive evaluations resulting from the mere arm flexion (Experiments 3 and 4).

Although cultures may vary endlessly in specific gestures associated with honesty or their opposites, our guess is that all such gestures may influence morally-relevant behavior, and perceptions when based on the same mechanism which we showed here for one particular gesture (hand-over-heart) in one particular culture (Poland). Of course, the meaning of the hand-over-heart gesture is culturally specific, although the fragment of Tony Blair’s speech cited at the beginning of the manuscript suggests that the British (in addition to the Poles who participated in our studies) also share the honesty-related meaning of the gesture. Probably, in an American context, raising one’s right hand (as one does in court when taking an oath to tell the truth, for example) would have a similar effect of increasing honesty. On the other hand, there are also some culturally-bound gestures associated with dishonesty rather than honesty. It is possible that American participants would lie more when crossing their fingers behind their backs, and would infer that a person displaying this gesture is less credible. In the same vein, French participants could express less confidence after fluttering their right hand with the palm down. Future research might also investigate principles underlying these gestures—for example, by differentiating between gestures that were culturally-learned and those that include biologically-predisposed bodily experiences.

One possible limitation of the current finding that people who put their hands over heart behave more honestly is that it may also be explained in terms of self-perception: because the gesture has a clear social meaning, people who display this gesture infer themselves to be honest and behave accordingly, perhaps due to an increased obligation to live up to a situationally-inflated standard of honesty. We argue, however, that self-perception processes do not provide a plausible explanation for the present data. Self-perception processes as conceptualized by Bem (1972) require conscious awareness of the meaning of the gesture. For example, studies on the self-perception of humor suggest that inferring affective states (feeling amused) from one’s own behavior (by smiling and laughing) requires awareness of the behavior in question, of its possible situational constraints (or lack of them), and most probably also an awareness of the relation between the two (Olson 1992). If induced by self-perception, any change in honesty due to the hand-over-heart gesture would require conscious attention to the gesture, recognition of its meaning, and awareness that the gesture has influenced the behavior. Our post-experimental interviews repeatedly showed no such awareness of these facts. Second, self-perception processes influence inferences of one’s own states or attributes to the degree that the latter are weak or ambiguous; this has been postulated by Bem (1972) and evidenced by others (e.g., Chaiken and Baldwin 1981). However, people’s convictions in their own honesty are anything but weak and ambiguous. For example, a great majority of the participants of one multi-sample study (*N* > 800 including pupils, students, and employees), showed quite extreme estimates of their honesty and other moral traits (Wojciszke et al. 2011), and extremity of ratings typically goes hand-in-hand with their subjective certainty. Third, the first two of the present studies showed the perception of others to be influenced by the hand-over-heart gesture, and by definition, self-perception processes cannot explain the perceptions of other persons. All of this suggests that self-perception processes do not provide a plausible explanation for the present data.

### Implications

Our results have several implications concerning both the domain of morality and embodiment literature. Our findings suggest that moral actions may be automatically triggered by incidental cues. Most prior research on impulsive antecedents of morally-relevant behavior have focused on how incidental cues increase immoral behavior, with the assumption that deliberative processes are necessary to enforce moral behavior. However, the logic of the impulsive system functioning assumed by the Reflective Impulsive Model (Strack and Deutsch 2004) extends equally well to negatively- and positively-valenced behavior. So, our results may be seen to fill an important gap in the empirical support for the idea of the impulsive regulation of morally-relevant behavior.

The present findings also have substantial implications for embodied perspectives on psychology. Grounded theories of cognition frequently account for the effects of bodily manipulation in terms of basic (good-bad) affective processes. In effect, embodied models often overlap with models predicting affective influences on judgments and behavior (Förster and Friedman 2008; Förster and Strack 1997; Parzuchowski and Szymkow-Sudziarska 2008; Petty and Cacioppo 1983; Strack et al. 1988). Our results extend this knowledge on embodiment by going beyond simple physical movements and global-affective reactions as dependent variables. Non-affective, bodily-primed concepts can trigger not only simple motor responses, but also complex goal-directed behavior, which may take different specific forms depending on circumstances. Providing frank estimates of the attractiveness of one’s friends, and refraining from cheating on a task each involve very different behavioral responses. What the two have in common is their abstract meaning of honesty, and the present studies show that this meaning can be primed by performing the hand-over-heart gesture. Simple motor movements are capable of influencing not only other motor programs, but also the abstract meaning of intentional behavior. Consequently, we would expect that various other behavioral expressions of honesty, such as the manifestation of prejudice (Wilson et al. 2000), the exertion of self-control when lying (Vohs et al. 2005), or suggestibility when implemented with false memories (Gheorghiu et al. 2003), could also be affected by the activation of the hand-over-heart gesture, or any other gesture defined culturally as an embodiment of honesty.

In summary, moral intuitions are not all about good and bad (see Haidt and Kesebir 2010). Our results suggest that associative non-affective cues can influence moral judgments (and behavior). This implies that the category of moral intuitions (or their antecedents) may be broader than previously thought. Also extending the work by Lakoff and Johnson (1980) on metaphor comprehension—an abstract concept of honesty—can be grounded on a very concrete level, and can be primed with an unobtrusive use of bodily feedback from a hand configuration. These findings suggest that the modal perceptual symbols that compose our knowledge of the honesty concept involve, among other things, a pattern of specific muscle activation that is used to signal sincere intentions with a hand-over-heart gesture.
